# Regulation of Postharvest Tomato Fruit Ripening by Endogenous Salicylic Acid

**DOI:** 10.3389/fpls.2021.663943

**Published:** 2021-06-07

**Authors:** Chunoti Changwal, Tushita Shukla, Zakir Hussain, Neera Singh, Abhijit Kar, Virendra P. Singh, M. Z. Abdin, Ajay Arora

**Affiliations:** ^1^Division of Plant Physiology, ICAR - Indian Agricultural Research Institute, New Delhi, India; ^2^Department of Biotechnology, Faculty of Science, Center for Transgenic Plant Development, Jamia Hamdard, New Delhi, India; ^3^Division of Vegetable Sciences, ICAR - Indian Agricultural Research Institute, New Delhi, India; ^4^Division of Agricultural Chemicals, ICAR - Indian Agricultural Research Institute, New Delhi, India; ^5^Division of Post-harvest Technology, ICAR - Indian Agricultural Research Institute, New Delhi, India

**Keywords:** ethylene, postharvest, salicylic acid, ripening, shelf life, tomato, *Isochorismate synthase*, phenylalanine ammonia-lyase

## Abstract

Exogenous application of salicylic acid (SA) has been known for delaying ripening in many fruit and vegetables. But the function of endogenous SA in relation to postharvest fruit performance is still unexplored. To understand the role of endogenous SA in postharvest fruit ripening of tomato, 33 tomato lines were examined for their endogenous SA content, membrane stability index (MSI), and shelf life (SL) at turning and red stages of tomato fruit ripening. Six tomato lines having contrasting shelf lives from these categories were subjected further for ethylene (ET) evolution, 1-aminocyclopropane-1-carboxylic acid synthase (ACS), 1-aminocyclopropane-1-carboxylic acid oxidase (ACO), polygalacturonase (PG), pectin methyl esterase (PME), antioxidant assays and lipid peroxidation. It was found that high endogenous SA has a direct association with low ET evolution, which leads to the high SL of fruit. High lycopene content was also found to be correlated with high SA. Total antioxidants, PG, and PME decreased and lipid peroxidation increased from turning to red stage of tomato fruit development. Furthermore, these lines were subjected to expression analysis for SA biosynthesis enzymes *viz. Solanum lycopersicum Isochorismate Synthase* (*SlICS*) and *SlPAL*. Real-time PCR data revealed that high SL lines have high *SlPAL4* expression and low SL lines have high *SlPAL6* expression. Based on the results obtained in this study, it was concluded that endogenous SA regulates ET evolution and SL with the aid of the antioxidative defense system, and *SlPAL4* and *SlPAL6* genes play significant but opposite roles during fruit ripening.

## Highlights

- Postharvest tomato fruit ripening depends on optimum concentrations of endogenous salicylic acid.
- Endogenous salicylic acid showed an association with postharvest ethylene evolution and shelf life of tomato fruit.
- Lycopene content in tomato fruit is negatively correlated with postharvest ethylene.
- *PAL* is involved in salicylic acid-mediated regulation of postharvest ripening.

## Introduction

Tomato is cultivated in outdoor settings, greenhouses, and net houses in nearly every country in the world. China, India, USA, Turkey, Egypt, Iran, Spain, Italy, and Brazil are the world's leading producer of tomatoes. It covers an area of ~4.73 million hectares and produces 163.96 million tons (FAO, 2016). After potato and onion, it is the world's third largest plant in production. Tomato is India's most common crop in terms of revenue and nutrition; it is a very valuable fruit plant. Tomatoes are primarily summer plants but can be grown year-round. Tomato is a climacteric fruit; its commencement of ripening is directly correlated to ethylene (ET) burst (Gapper et al., 2013). To deal with the postharvest losses due to early onset of ripening and associated changes, ET synthesis needs to be either blocked or slowed down. So, it is very important to study every component that may affect the ET production. In most of the fruits including tomato, there is a change in color, fruit firmness, electrolyte leakage, etc., which indicates fruit maturation. Tomato undergoes six developmental stages of fruit ripening: green, breaker, turning, pink, light red, and red ripe. Tomato fruit ripening is escorted by fruit softening, changes in color from green to red, and increased levels of flavor and aromatic compounds such as sugars, volatiles, and organic acids.

The increasing demand for fresh vegetables and fruit along with less use of synthetic chemicals to reduce postharvest losses has encouraged research groups to develop technologies based on natural molecules, such as salicylic acid (SA). SA is an endogenous phenolic growth regulator involved in the regulation of various processes in plant, such as stomata movement, ion absorption, induction of disease resistance, sex polarization (Raskin, 1992), leaf senescence (Morris et al., 2000), flowering (Martínez et al., 2004), seed germination (Xie et al., 2007), regulation of root water transport (Quiroga et al., 2018); abiotic stresses such as easing heat stress (Larkindale and Knight, 2002**)**, alleviating heavy metal toxicity in tomato (Songül Çanakci and Dursun, 2012), reducing chilling injury (Aghdam et al., 2012; Goyal et al., 2016), increasing salt tolerance (Gharbi et al., 2018), improving freezing tolerance (Shin et al., 2018), etc. SA can exist in two forms: free SA and conjugated SA. SA conjugates with various molecules either by esterification or by glycosylation (Popova et al., 1997). SA application causes overproduction of different forms of reactive oxygen species (ROS) and nitrogen species causing activation or repression of many signaling pathways (Gémes et al., 2011; Jayakannan et al., 2015). ROS, such as hydrogen peroxide, hydroxyl ions, and superoxide, cause oxidative damage at the cellular level (Goud and Kachole, 2011). ROS may play two different roles *viz*. activating the defense response system or exacerbating damage. In low concentrations, ROS work as signaling molecules mediating various physiological responses, including stomatal movement and gene (Suzuki et al., 2012; Yi et al., 2014). But high concentrations of ROS degrade cell membrane lipids, enzymes, chloroplasts, pigments, and nucleic acids (Goud and Kachole, 2011). To overcome this, plants have evolved various mechanisms to survive environmental stresses. For example upregulation of enzymatic antioxidants [such as peroxidase (POX), superoxide dismutase (SOD), and catalase (CAT); Noctor and Foyer, 1998], non-enzymatic metabolites (such as ascorbic acid), and osmoprotectants (Farooq et al., 2008; Gautam and Singh, 2009). The final effect of SA on plant depends on its concentration, plant type, plant growth stage, and environmental conditions (Miura and Tada, 2014). In broad terms, when applied externally, low SA concentrations increase the antioxidant power and tolerance to abiotic stresses, but high SA concentrations may lead to abiotic stress susceptibility or cell death (Hara et al., 2012).

There are plenty of reports showing that preharvest or postharvest application of SA delays the ripening of fruit and vegetables such as banana (Srivastava and Dwivedi, 2000), tomato fruit (Ruifen, 2001; Mandal et al., 2018), kiwi (Zhang et al., 2003), peach (Han et al., 2002; Tareen et al., 2012), strawberry (Lolaei et al., 2012), mango (Hong et al., 2014), etc. SA probably delays ripening by inhibition of ET biosynthesis at the level of 1-aminocyclopropane-1-carboxylic acid (ACC) (Leslie and Romani, 1988). Recently, it has also been reported that addition of SA inhibits 1-aminocyclopropane-1-carboxylic acid synthase (ACS1) and 1-aminocyclopropane-1-carboxylic acid oxidase (ACO1) in rice (Zhu et al., 2020). It also trimmed down the quality loss in tomato (Ding et al., 2001), sweet pepper (Fung et al., 2004), loquat (Cai et al., 2006), peach (Wang et al., 2006), mango (Ding et al., 2007), pomegranate (Sayyari et al., 2011), and mangosteen (Mustafa et al., 2018). As the fruit detaches from the plant, its whole physiology changes so ripening on-vine and ripening off-vine are two different developmental processes (Paul et al., 2012). The role of SA, auxin, and melatonin in climacteric and non-climacteric fruit ripening has recently been reviewed by Pérez-Llorca et al. (2019). But the endogenous SA and its association with postharvest ripening need more study. Though some research groups such as Blanco-Ulate et al. (2013) have done some work on studying the interaction of intrinsic SA with other plant hormonal genes during on-vine ripening and pathogen infection, research work aiming at endogenous SA and its interaction with fruit ripening after the harvest is obscure. Keeping in view this gap in literature, we attempted to determine the endogenous SA content across the tomato germplasm and establish its relationship with various physiological and molecular postharvest traits related to fruit ripening. We try to decipher how the change in inherent (on-vine) endogenous SA concentration in different tomato varieties/hybrids affects the off-vine ripening and postharvest parameters. So, here, we hypothesize that just like exogenous SA, endogenous SA of a plant does play a role in postharvest ripening.

## Materials and Methods

### Plant Material

Tomato (*Solanum lycopersicum* L.) fruit was used in this study. Thirty-three tomato fruit lines (Supplementary Table 1) were obtained from the Research Farm of Division of Vegetable Sciences, ICAR-IARI, New Delhi. These plants were grown in the month of November in winter (Rabi) season at 23 ± 2°C, relative humidity (RH) 60%, and fruits were harvested during April–May with temperatures ranging from 27 to 35°C and RH 50–60%. Tomato fruits were harvested at mature green (full light green to dark green color), turning (10–30% of color change), light red (60–90% of color change), and red (more than 90% of color change) stages of tomato fruit ripening according to “color classification requirements in United States standards for grades of fresh tomatoes.” Fruits of uniform size were selected at each sampling time during 2 years of sampling, and in every experiment, three replications per lines were used. The fruit pericarp was frozen in liquid nitrogen and stored at −80°C for different experiments.

### Salicylic Acid Content in Tomato Fruit

For evaluation of SA content in tomato fruit, the fruits of green, turning, and red stages were harvested. SA content was analyzed through high-performance liquid chromatography (HPLC). SA extraction protocol from tomato pericarp was adapted from O'Donnell et al. (2001) with some modifications as described further. Six-gram tissues of tomato pericarp were crushed in liquid N_2_ and extracted in 15 ml of 90% methanol followed by 10 ml of 100% methanol by centrifugation at 10,000 g for 15 min. The combined extracts were divided into two and dried down separately by rotary evaporator. For free SA determination, residues were resuspended in 1.5 ml 100% methanol and subjected to HPLC analysis. For conjugated SA, residues were hydrolyzed in 2 M HCl for 60 min at 65°C, and the resulting fractions were then extracted twice in diethyl ether, dried down, and resuspended in methanol. SA was quantified by reverse-phase HPLC (Varian, Prostar, Santa Monica, CA, USA) equipped with quaternary pump, UV detector and connected with Rheodyne injection system using Lichrospher C-18 stainless steel column (4 mm × 250 mm i.d.), acetonitrile:0.1% aqueous o-phosphoric acid (40:60) as a mobile phase at a flow rate of 1.0 min^−1^ at wavelength of 230 nm. The recovery of SA was estimated by extracting tissue to which a known amount of SA has been added. The results were estimated from the graph made by running the standard (Sigma-Aldrich, India) of different concentrations.

### Real-Time Quantitative RT-PCR

RNA was extracted from tomato fruit of green, turning, and red stages manually using RNAiso Plus reagent (Takara, Japan), followed by a DNA elimination step. First-strand cDNA synthesis was conducted using the Super Script First Strand Synthesis kit (Invitrogen, Carlsbad, CA, USA). From NCBI and TIGR databases, cDNA sequences of *Solanum lycopersicum Isochorismate Synthase (SlICS*) and six tomato *Solanum lycopersicum Phenylalanine Ammonia Lyase (SlPAL)* genes that differed in their non-coding 3' ends were determined. The primer pair for the *SlICS* was designed using primer3 software. Primers for *SlPAL* genes and the reference genes were picked up from previous studies (Table 1) (Løvdal and Lillo, 2009; Gayoso et al., 2010). Real-time RT-PCR was performed in 25 μl reaction mixture composing 0.2 mM of each primer and 1x SYBR Green supermix (Kapabiosystems, Massachusetts, USA) using CFX96 RT-PCR system (BioRad, UK). The thermal cycling conditions for all the genes consisted of an initial denaturation step at 95°C for 3 min followed by 40 cycles at 95°C for 10 s and 60°C for 20 s and a final step at 72°C for 5 min. For the comparison of levels of expression among genes at different ripening stages of fruit, the gene expression at the green stage was taken as calibrator. Three replicates were maintained per reaction, and a negative template control was used for every run. The threshold cycle values were normalized by β*-TUBULIN* as endogenous control, and fold changes of the target gene were calculated by 2^−ΔΔCt^ method (Livak and Schmittgen, 2001).

**Table 1:** Primers used in RT-qPCR gene expression analysis.

**Gene**	**Forward primer sequence (5′ to 3′)**	**Reverse primer sequence (5′ to 3′)**
β-tubulin	AACCTCCATTCAGGAGATGTTT	TCTGCTGTAGCATCCTGGTATT
GAPDH	GGCTGCAATCAAGGAGGAA	AAATCAATCACACGGGAACTG
*SlICS*	GAAATGTTTGACCGAGGAATG	CCCAGTTCCCGCATAAAT
*SlPAL4*	CGGTGAGGAGATTGACAAGG	CCTGTAAAGTTGTAGAAATTGAATGAA
*SlPAL6*	TTGCAAACAGGATCAACGAA	TTGCTTCACTTCACTTCTAACAGACTGG

*GAPDH, Glyceraldehyde 3-Phospate Dehydrogenase; SlICS, Solanum lycopersicum Isochorismate Synthase; SlPAL, Solanum lycopersicum Phenylalanine Ammonia Lyase*.

### Determination of Ethylene Evolution

For ET estimation, fruits were picked at mature green stage in three replicates and kept at 25°C for 2 h to reduce harvest shock. They were then weighed and individually placed in an airtight container equipped with a rubber stopper for 1 h at 25°C. One milliliter of headspace gas was taken for ET determination and injected into a gas chromatograph (GC; Hewlett Packard series II, Wipro, GC 5890, USA) fitted with a flame ionization detector and stainless steel porapack column. For the estimation of postharvest ET at subsequent developmental stages, fruits were kept at 25°C room temperature to achieve the required stage. ET evolution was expressed in μg kg^−1^ s^−1^. The measurement conditions were: 5 ft stainless steel porapack column; the temperature of column, injector, and detector was 60, 62, and 250°C, respectively. Nitrogen was used as the carrier gas, and an ET standard of 52.843 mg L^−1^ was used.

### Extraction and Assay of 1-Aminocyclopropane-1-Carboxylic Acid Synthase and 1-Aminocyclopropane-1-Carboxylic Acid Oxidase Activity

ACS and ACO activities were determined according to Khan and Singh (2007). For ACO activity, tomato fruit pericarp (2 g) was homogenized in pestle and mortar in 5 ml extraction buffer consisting of 0.1 M Tris-HCl (pH 7.2), 10% (w/v) glycerol, and 30 mM sodium ascorbate in the presence of 5% polyvinylpyrrolidone (PVP), and the mixture was centrifuged at 12,000 g for 30 min at 4°C. The enzyme was assayed in 2 ml reaction mixture containing 1.8 ml of the above supernatant solution, 0.1 ml ACC (40 mM), and 0.1 ml FeSO_4_ (1 mM). The reaction tube was sealed with a rubber septum and incubated at 30°C for 60 min. One milliliter of gas was taken from headspace and injected into GC for ET estimation, as described earlier.

For ACS activity, tomato fruit (10 g) was homogenized with 5 ml K-phosphate buffer (0.5 M, pH 8.5) having 5 μM pyridoxal phosphate and 5 mM dithiothreitol and of 5% PVP in mortar and pestle at 4°C. Contents were centrifuged at 12,000 g for 30 min at 4°C, and 2 ml of supernatant was mixed with 1 ml of 500 μM s-adenosylmethionine (SAM; Sigma Aldrich, India). The reaction tube was sealed with a rubber septum, incubated for 30 min at 30°C, and transferred to an ice bath. Using a syringe through the stopper, 0.1 ml of HgCl_2_ (50 mM) and 0.3 ml of NaOCl (5%) and saturated NaOH (2:1, v/v) were added into the reaction tube. The reaction tube was incubated in ice for further 2.5 min, and a 1-ml gas sample was taken from the headspace and injected into the GC for ET estimation, as described earlier. The activity was expressed in micrograms per kilogram of protein per second (μg kg^−1^ s^−1^). The protein level was determined using the Bradford method (Bradford, 1976) using bovine serum albumin as a standard.

### Determination of Fruit Shelf Life and Electrolyte Leakage

Fruits were harvested at the mature green stage and allowed to ripen at room temperature (25 ± 2°C). Days were calculated from mature green stage until the first sign of shriveling as fruit shelf life (SL). The data were calculated for 2 years in six replications every time.

Membrane stability index (MSI) was measured as a function of electrolyte leakage, which was estimated using an EC 215 conductivity meter (Hanna Instruments, Padova, Italy), as described by Sairam (1994). Fruit tissues were incubated in distilled water at 40°C for 30 min to determine the initial electrolyte leakage (Ei), and it was considered as control. The tubes were then boiled in water for 15 min, cooled to room temperature, and the final electrolyte leakage (Ef) was measured. The following formula was used to calculate the relative electrolyte leakage:

$$\begin{array}{l} \text{MSI}\%=\left(1-\frac{Ei}{Ef}\right)100 \end{array}$$

Three fruit replicates were maintained per stage for each line.

### Assay of Cell Wall Softening Enzymes

Polygalacturonase (PG) activity was determined according to Jhalegar et al. (2012). For it, 5 g fruit tissue was grounded in 10 ml of 0.2 M sodium acetate buffer (pH 6) along with a pinch of Na_2_S_2_O_4_ and a pinch of PVP at 4°C in ice. Then, this homogenate was centrifuged at 15,000 g for 20 min at 4°C, the supernatant was collected, and this enzyme extract was used for both PG and pectin methyl esterase (PME) assay. For PG, assay mixture consisted of 0.45% (w/v) pure pectin (SRL, India), 0.1% casein in 0.4% sodium acetate buffer (pH 3.8). Here, 0.2 ml of assay mixture was added to 2 ml enzyme extract and incubated in a water bath at 37°C for 2 h. To 0.05 ml of this incubated enzyme extract, 5 ml of 96% H_2_SO_4_ was added and allowed to react for 15 min. The blank contained 0.05 ml distilled H_2_O instead of enzyme extract. Then, 5 ml of distilled H_2_O was added; the mixture was vortexed and cooled to room temperature. Optical density (OD) was measured at 490 nm. PG activity was calculated as milligrams of glucose equivalent released per kilogram fresh weight per second (mg kg^−1^ s^−1^). For PME, the enzyme extract of PG was used. PME assay was adapted from Giovane et al. (1996) and Carvalho et al. (2009) with some modification and optimization. Assay mixture for PME consisted of 0.3 mM NaCl (pH 8) and 0.5% pure pectin (SRL, India). Here, 10 ml of assay mixture was added to 3 ml of enzyme extract gradually with constant stirring. After 5 min, the pH of the mixture was brought to the original pH value (pH 8) with 0.1 M NaOH. The volume of 0.1 M NaOH consumed was noted down. PME activity was expressed in unit equivalents per second per kilogram fresh weight (s^−1^ kg^−1^) = 0.13 × volume of 0.1 M NaOH consumed.

### Lipid Peroxidation

Lipid peroxidation is oxidative degradation of lipid-fatty acids by ROS. The level of lipid peroxidation is measured in terms of thiobarbituric acid reactive substances (TBARS) content. It was evaluated by the reaction of one of the lipid peroxidation products, malondialdehyde (MDA) with TBARS, as described by Heath and Packer (1968). Results were expressed as nanomoles per kilogram of fresh weight (nmol kg^−1^).

### Ascorbic Acid Content

Ascorbic acid was determined by dinitrophenylhydrazine reagent method as described by Mukherjee and Choudhuri (1983). Results were expressed as grams per kilogram on fresh weight basis (g kg^−1^).

### Assay of Superoxide Dismutase

SOD was measured according to Dhindsa et al. (1981). Results were expressed as millimoles per kilogram per second (mmol kg^−1^ s^−1^).

### Ascorbate Peroxidase

Ascorbate peroxidase (APX) was assayed as described by Nakano and Asada (1981). Enzyme activity is calculated as the concentration of ascorbic acid oxidized per kilogram protein per second (mmol kg^−1^ s^−1^).

### Glutathione Reductase

Glutathione reductase (GR) was measured according to Smith et al. (1988b). The absorbance was taken at 412 nm and the activity is expressed as moles per kilogram protein per second (mol kg^−1^ s^−1^).

### Catalase

CAT was determined according to Aebi (1984). The activity is expressed as the concentration of hydrogen peroxide reduced per kilogram protein per second (mol kg^−1^ s^−1^).

### Peroxidase

POX was calculated as the increase in OD due to oxidation of guaiacol to tetraguaiacol as described by Castillo et al. (1984). Enzyme activity is expressed as mol of tetraguaiacol formed per kilogram protein per second (mol kg^−1^ s^−1^).

### Lycopene Content

Lycopene is the major membrane-bound antioxidant in mature red tomato fruit. Lycopene from tomato fruit was extracted with hexane:methanol:acetone (2:1:1) containing 2.5% butylated hydroxy toluene (BHT). The OD of the hexane extract was measured at 502 nm against a hexane blank. The concentration of lycopene was calculated using the extinction coefficient (E) of 3,150 (Rao et al., 1998). Results were expressed as gram lycopene per kilogram of fresh weight (g kg^−1^).

### Total Antioxidant

The total antioxidants of fruit were determined using the ferric reducing ability of plasma (FRAP) method by Benzie and Strain (1999) as a measure of antioxidant power. FRAP value was expressed as mol FeSO_4_ per kilogram of fresh weight (mol kg^−1^).

### Statistical Analysis

All experiments were done in triplicate unless otherwise mentioned, and the results were expressed as means ± standard error (SE). The data were subjected to one-way or two-way analysis of variance (ANOVA) using SPSS version 16 (IBM Corp., Armonk, NY, USA), where differences of *P* < 0.05 were considered significant. A Pearson correlation matrix was used to calculate the correlation coefficient (r) between all parameters. Cluster analysis was done by R software version 3.6.1.

## Results

**Figure 1:**
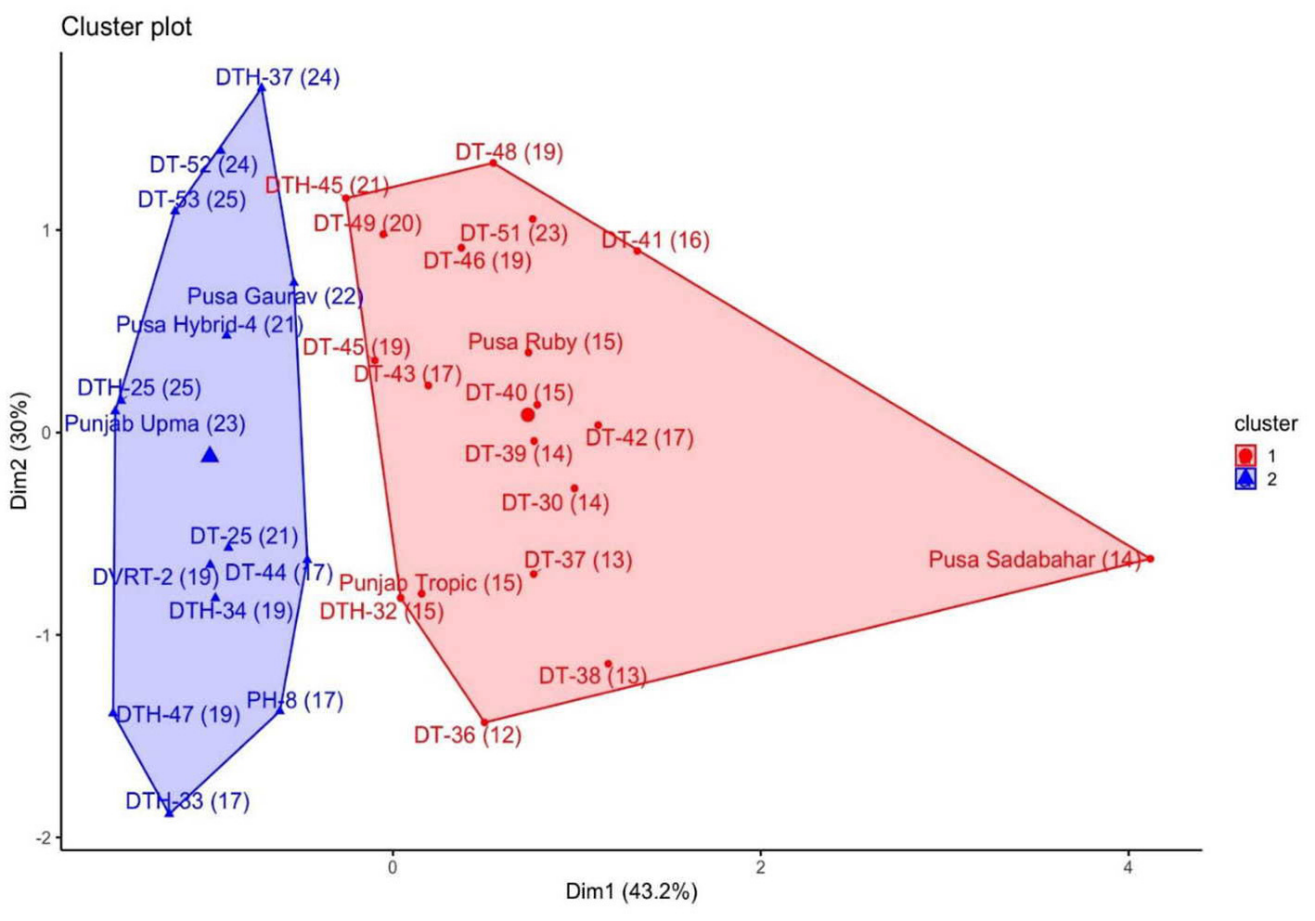
Groups made by K-mean clustering method of all 33 lines. The blue cluster represents all high shelf life lines.

To study the role of endogenous SA in the postharvest ripening of tomato fruit, a mix of 33 tomato varieties/cultivars/pure lines/breeding lines/hybrids (denoted as lines from now on) was evaluated in field conditions of ICAR-IARI, New Delhi, India (Supplementary Table 1). These lines were grouped into two clusters generated by K-mean cluster analysis (Figure 1) based on endogenous SA, electrolyte leakage, and SL. The SL of lines ranged from 12 to 25 days, and we categorized these fruits into two groups: one is “Low shelf life” (12–16 days SL) fruit and another is “High shelf life” (17–25 days SL). Among the two clusters, at turning stage, one cluster was composed of nine low SL and 10 high SL lines, and the other cluster was composed of all high SL lines (14).

From the first cluster, three low SL lines *viz*. DT-30, Punjab Tropic, and Pusa Ruby and, from the second cluster, three high SL lines *viz*. DT-25, Pusa Gaurav, and DT-52 were chosen as contrasting lines for further studies to work out the relationship between endogenous SA and postharvest ripening. So, a total of six tomato lines were selected (three from each cluster) for further experimentations. These lines were selected (apart from contrasting SLs) due to the following characteristics: (1) DT-30 is a pre-released pure line of importance; (2) Pusa Ruby and Pusa Gaurav are very well-known Indian varieties having contrasting SLs; (3) DT-52 and Punjab Tropic are well-cultivated Indian variety/cultivar having commercial importance; (4) DT-25 is a breeding line having quality impact.

### Endogenous Salicylic Acid Content and Postharvest Ethylene Evolution in Fruits at Different Ripening Stages

**Figure 2:**
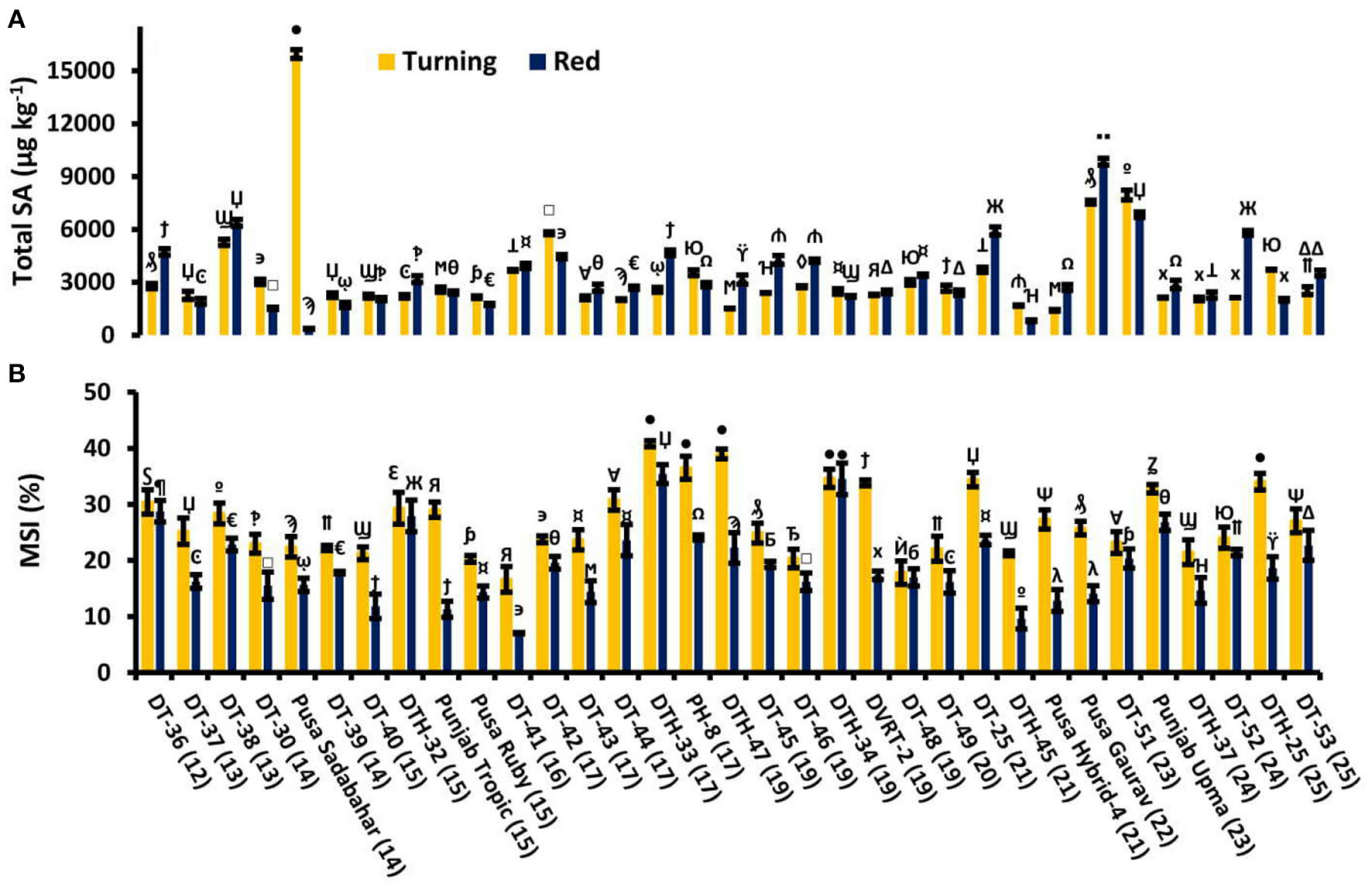
Tomato lines with total salicylic acid (SA) at turning stage and the red stage of tomato fruit development **(A)** with their respective membrane stability index (MSI) **(B)**. Bottom axis represents the name of tomato lines, and numbers enclosed in parentheses represent shelf life. Each graph represents the mean of three replications, and vertical bars represent ±SE. Different symbols over the bars represent the significant differences at *P* < 0.05.

We studied free, conjugated, and total SA in 33 lines at two tomato fruit developmental stages, turning and red. Some tomato lines showed decreased and others were observed to be having increased values of SA (Figure 2A). Furthermore, electrolyte leakage was also measured, which showed a consistent decrease in MSI from turning to red stages in all the fruits studied (Figure 2B).

**Figure 3:**
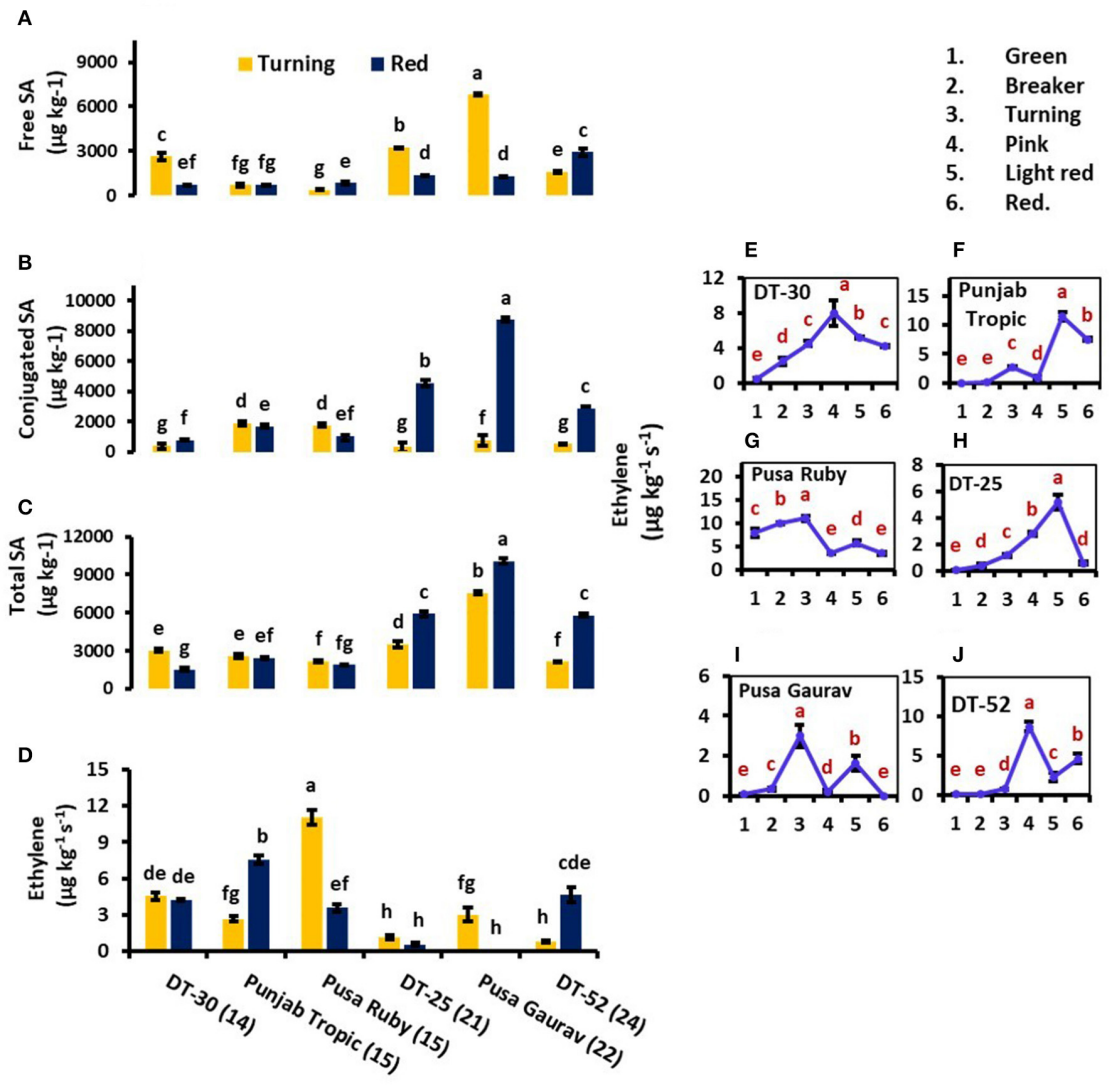
Comparison of free salicylic acid (SA) **(A)**, conjugated SA **(B)**, total endogenous SA **(C)**, and ethylene (ET) evolution **(D)** at turning and red stages of tomato fruit development. Bottom axis represents the name of tomato lines, and numbers enclosed in parentheses represent shelf life. ET evolution from mature green to red stage of individual tomato fruit line **(E–J)**. Each graph represents the mean of three replications, and vertical bars represent ±SE. Some error bars are hidden behind marker. Different letters represent significant differences at *P* < 0.05.

To better understand the relationship of intrinsic SA and postharvest ET release, concentration factor (CF) was also calculated, which is a ratio of SA and ET (Table 2). At turning stage, CF ranged from 190 to 925 in low SL lines and 930 to 3,100 in high SL lines. While at red stage, CF from 300 to 600 was calculated in low SL and more than 1,200 in high SL lines. SA was found to be high and corresponding ET evolution to be low in high SL lines (Figures 3B–D).

**Table 2:** Concentration factors of the tomato fruits.

**Lines**	**Shelf life**	**Concentration factor (total SA/ET)**	
		**Turning stage**	**Red stage**
DT-30	14	718.54	361.08
PUNJAB TROPIC	15	923.46	335.22
PUSA RUBY	15	195.78	523.25
DT-25	21	3,071.31	10,184.50
PUSA GAURAV	22	938.33	36,474,072.73
DT-52	24	2,736.40	1,240.00

At red stage, the conjugated and total SA showed a prominent rise in SA content in only high SL lines *viz*. DT-25, Pusa Gaurav, and DT-52 (Figures 3B,C); however, free SA did not show any definite pattern in all the six lines studied (Figure 3A). These results indicated that endogenous SA regulates ET evolution more prominently at later stages of fruit ripening, while in initial stages, the plants try to acclimatize. Again, at red stage, total endogenous SA showed a negative correlation with the rate of ET evolution (*P* = 0.05; *r* = −0.71) and positive with SL (*P* < 0.05; *r* = 0.829), while free SA had a strong positive correlation with SL (*P* < 0.05; *r* = 0.851), *PAL4*, CAT, and POX (Table 3).

**Table 3:** Linear correlation between total endogenous SA content and other parameters at turning and red stages of tomato fruit ripening at *P* < 0.05.

**Parameter**	**Total SA (Turning)**	**Total SA (Red)**	**Free SA (Turning)**	**Free SA (Red)**
ET	−0.210	−0.710	−0.359	−0.100
Shelf life	0.357	0.829^*^	0.467	0.851^*^
MSI	0.256	0.889^*^	0.320	0.689
*SlICS*	−0.194	0.957^**^	−0.400	0.693
*SlPAL4*	0.758	0.193	−0.838^**^	0.940^*^
*SlPAL6*	−0.356	−0.359	−0.467	−0.149
ACO	–	−0.293	–	0.079
ACS	–	−0.639	–	−0.481
PG	−0.332	−0.222	−0.228	0.765
PME	0.497	0.528	0.578	−0.409
FRAP	0.027	−0.552	0.144	−0.252
SOD	−0.268	0.044	−0.094	0.153
AA	−0.639	0.101	−0.654	0.036
Lycopene	–	0.625	–	−0.046
CAT	−0.128	0.074	0.110	0.809^*^
POX	−0.443	0.383	−0.186	0.969^**^
GR	−0.504	0.764	−0.335	0.735
APX	0.058	0.064	−0.432	−0.042
TBARS	−0.395	0.015	−0.177	0.567

** *Correlation is significant at the 0.01 level (two-tailed)*.

* *Correlation is significant at the 0.05 level (two-tailed)*.

### Ethylene Biosynthesis in Fruit

**Figure 4:**
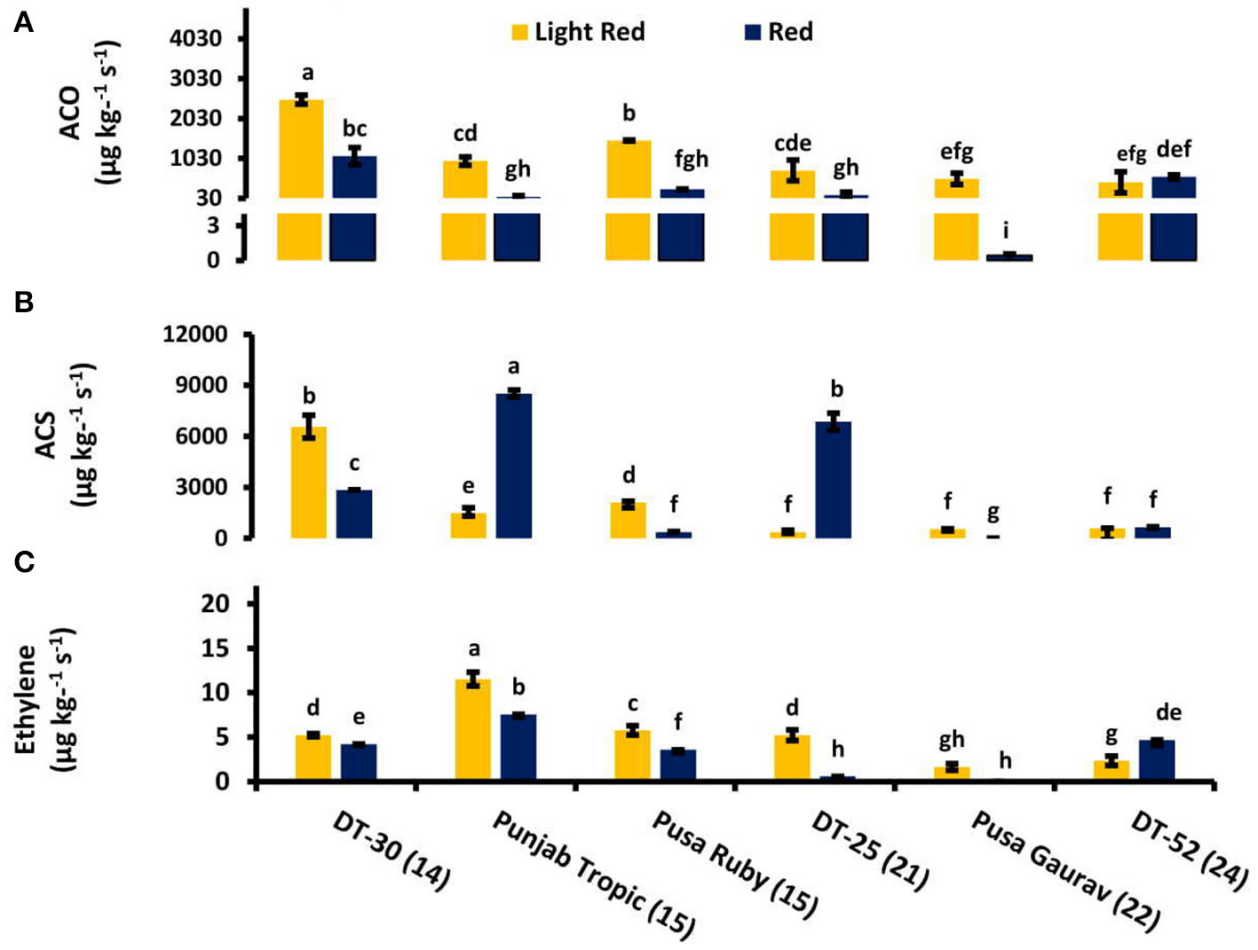
Comparison of **(A)** 1-aminocyclopropane-1-carboxylic acid (ACC) oxidase activity, **(B)** ACC synthase activity, and **(C)** ethylene (ET) evolution at light red and red stage. The bottom axis represents the name of tomato lines, and numbers enclosed in parentheses represent shelf life. Measurements were conducted in three replicates. Results are presented as means ± SE. Different letters represent significant differences at *P* < 0.05.

As we moved from mature green to red stage, ET evolution peaked at either turning or pink stage in all lines except DT-25, where it peaked at light red stage. However, in some lines, two ET peaks were also observed (Figures 3E–J). To relate ACO and ACS activities with postharvest ET evolution, two fruit developmental stages *viz*. light red and red were used. These stages were chosen because, in them, the concentration of the enzyme can be found at the detectable amount. In all the lines studied, ACO was observed to be working consistently with ET from turning to red stage. However, it is noteworthy that the level of ACS activity was higher than that of ACO (Figure 4).

### Expression of Endogenous Salicylic Acid Biosynthesis Enzymes During Ripening

**Figure 5:**
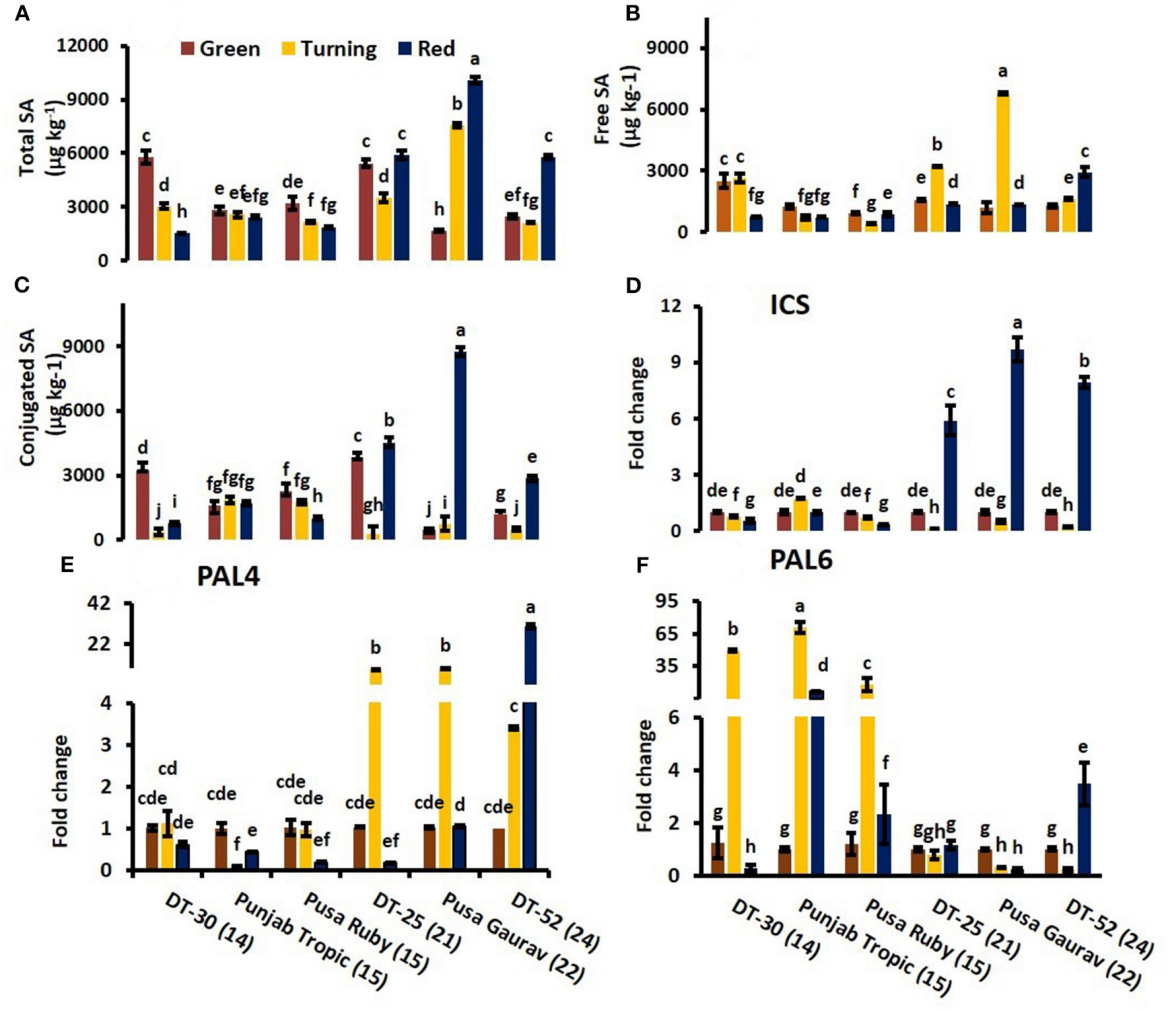
Relative expression of SA biosynthesis enzymes viz. *Isochorismate Synthase* **(D)**, *Phenylalanine Ammonia Lyase 4* **(E)** and *Phenylalanine Ammonia Lyase 6* **(F)** in relation to total endogenous SA **(A)**, free SA **(B)** and conjugated SA **(C)** level at turning and red stage. Bottom axis represents name of tomato lines and numbers enclosed in parenthesis represents shelf life. The expression data of green stage fruit were normalized to a value of 1. Each value represents the mean ± S.E. of three replicates. Different letters represent significant differences at *P* < 0.05.

To investigate the gene(s) involved in endogenous SA-mediated regulation of postharvest ripening, expressions of its biosynthetic pathway genes *viz. ICS* and *PAL* were analyzed. *ICS* has only one isoform in tomato, and expression analysis of *SlICS* revealed its synchrony with total endogenous SA content and with conjugated SA (especially in high SL lines) during fruit ripening (Figures 5A,C,D). At red stage, correlation analysis of *SlICS* with total SA was also found to be positive (*P* ≤ 0.001; *r* = 0.957), indicating the high association among them.

To determine tomato *SlPAL* and ripening relationship, expression of *SlPAL4* and *SlPAL6* were studied. *SlPAL6* showed a significantly high level of expression in low SL lines (Figure 5F), and *SlPAL4* showed upregulated expression in high SL lines (Figure 5E). At red stage, *PAL4* was found to be positively correlated with free SA (*P* ≤ 0.05; *r* = 0.940). It suggests that endogenous SA regulates ripening at the level of *PAL*, and *PAL4* is involved in SA-induced reduction of ripening (Figure 5B).

### Antioxidative Defense and Cell Wall-Degrading Enzymes

**Figure 6:**
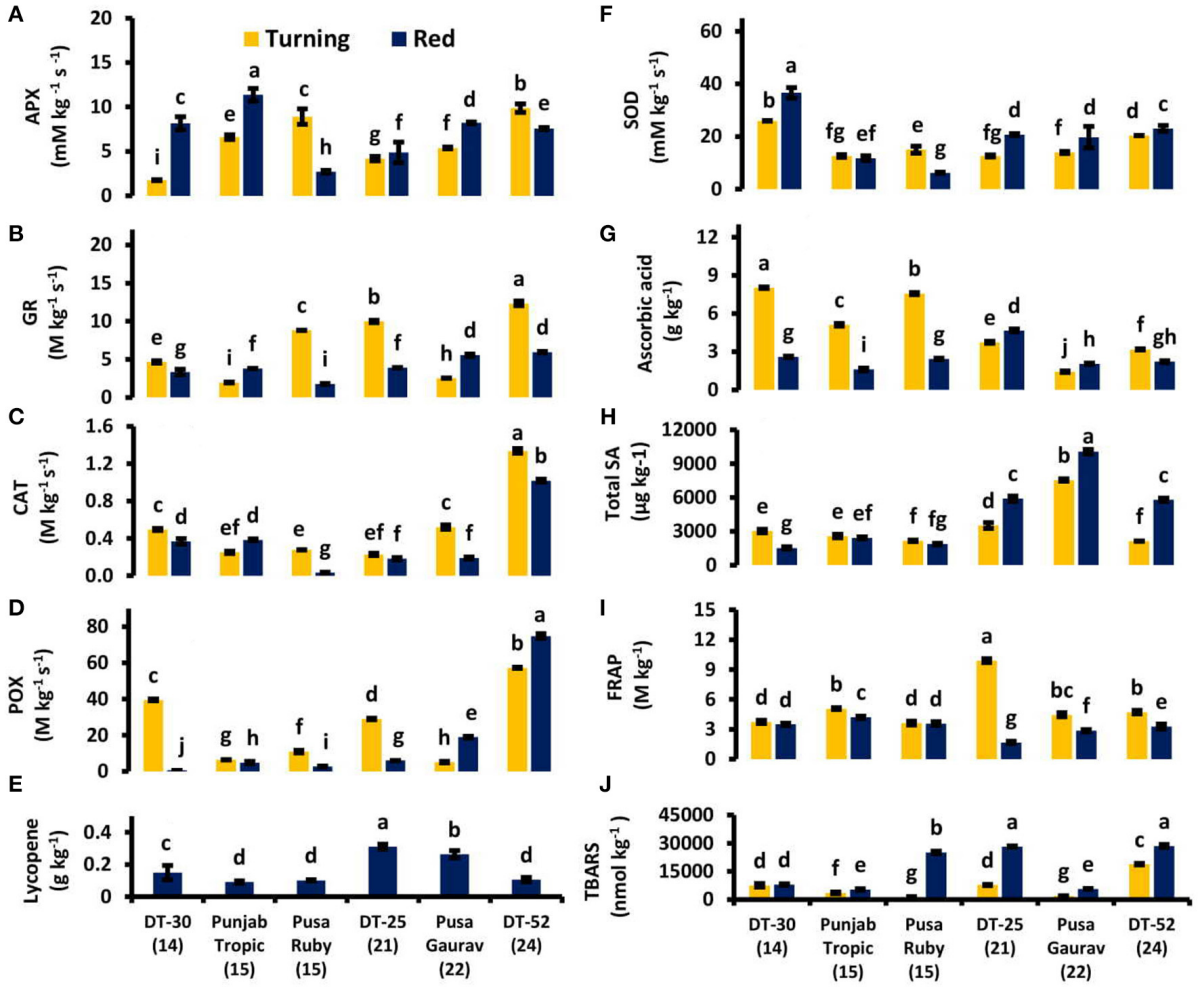
Ascorbate peroxidase (APX) **(A)**, glutathione reductase (GR) **(B)**, catalase (CAT) **(C)**, peroxidase (POX) **(D)**, lycopene **(E)**, superoxide dismutase (SOD) **(F)** activities and ascorbic acid content **(G)**, total endogenous salicylic acid (SA) **(H)**, total antioxidants by ferric reducing ability of plasma (FRAP) **(I)** and lipid peroxidation by thiobarbituric acid reactive substances (TBARS) **(J)** in tomato fruit at turning and red stages. Bottom axis represents the name of tomato lines, and numbers enclosed in parentheses represent shelf life. Each graph represents the mean of three replications, and vertical bars represent ±SE.

While studying the postharvest fruit ripening, quality of fruit is also a major factor to be considered. Antioxidative potential and cell wall rigidity are major attributes deciding quality and hence marketability of a fruit. So, after analyzing the fruit at the molecular level, we planned to study the relationship of endogenous SA with ROS metabolism. The total antioxidative capacity was observed to be decreasing from turning to red stages, while individual antioxidants showed different patterns that vary from lines to lines (Figure 6).

Correlation studies were performed to find out the impact of various antioxidants on postharvest fruit ripening. SOD was also found to be positively correlated with POX at turning stage (Supplementary Table 2). Ascorbic acid (AA) was observed to be high in low SL lines as compared to the high SL lines especially at the turning stage. A positive correlation was also found between conjugated SA and AA at red stage (Supplementary Table 3). Lycopene content was observed to be negatively correlated with ET evolution at red stage (*P* < 0.05; *r* = −0.870). The highest lycopene content was observed in DT-25 followed by Pusa Gaurav (Figure 6E).

The TBARS increased with the senescence, indicating an increased level of lipid peroxidation. Lipid peroxidation was found to be increased as we progress from turning to red stages in all the lines (Figure 6J). MSI has a positive correlation with SL at both stage and ICS at red stage (Supplementary Tables 2, 3).

**Figure 7:**
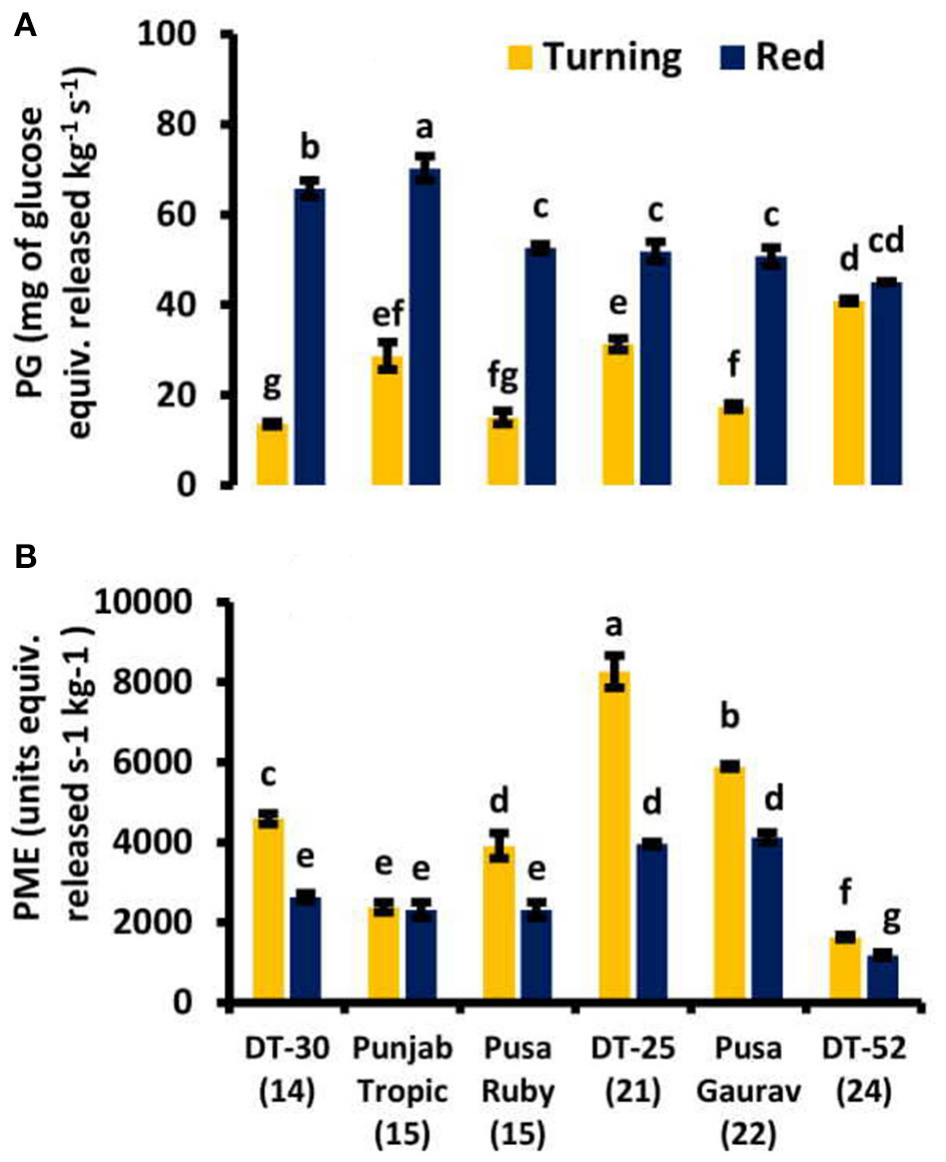
Cell wall softening enzymes *viz*. **(A)** polygalacturonase (PG) and **(B)** pectin methyl esterase (PME) activities in tomato fruit at turning and red stages. Bottom axis represents the name of tomato lines, and numbers enclosed in parentheses represent shelf life. Each graph represents the mean of three replications, and vertical bars represent ±SE.

In the present study, two cell wall-softening enzymes were selected for studying the effect on the SL of fruit *viz*. PG and PME. It was found that PG has an increasing pattern from turning to the red stage in all the lines, and PME was found to be decreasing from turning to red stages (Figure 7). PG is also positively correlated with free SA, CAT, and POX, while PME is positively correlated with lycopene and negatively with ET evolution at red stage (Supplementary Table 3).

## Discussion

Role of exogenous SA application in delaying ripening is a well-established phenomenon. But the function of endogenous SA *vis-à-vis* postharvest fruit performance is still unknown. So, it was hypothesized that variation in endogenous SA also contributes to modulate postharvest ripening. To find out its role, SA concentration of 33 tomato lines was measured and compared with various physiological and molecular traits such as SA biosynthesis genes, postharvest ET biosynthesis, cell wall softening, antioxidative defense, and oxidative stress of tomato fruit. It was found out that endogenous SA regulates ET. There are several reports of correlation studies between different attributes (such as various antioxidants) in many vegetable and fruits (Arias et al., 2000; Meléndez-Martínez et al., 2010; García-Valverde et al., 2013; Bhandari and Lee, 2016; Dinu et al., 2018). So, to understand the contribution of endogenous SA to postharvest ripening regardless of the tomato line, we performed a correlation analysis.

### Concentration-Dependent Association of Endogenous Salicylic Acid and Postharvest Ethylene Biosynthesis

ET plays a key role in fruit ripening and senescence by triggering the induction of cell wall hydrolyzing enzymes leading to fruit softening, increase in respiration rate, and senescence (Wills et al., 1998). Exogenous SA treatment proved to be a potent inhibitor of ET biosynthesis in several crops (Leslie and Romani, 1988; Romani et al., 1989; Srivastava and Dwivedi, 2000; Babalar et al., 2007; Aghdam et al., 2009). In the present study, the lower level of endogenous SA content in low SL lines and the higher level in high SL lines indicate that SA regulates ET evolution at a certain level (Figure 3, Table 3). In Pusa Gaurav, DT-25, and DT-52, the high SA and CF at red stage were sufficient enough to lower the ET and consequently increase the SL of fruit (Figure 3, Table 2). The variation in CF value is more prominent at red stage than at turning stage. This is a direct indication that, in initial stages, the plant tries to acclimatize itself toward its internal as well as external environment and endogenous SA regulates ET evolution significantly at later stages of fruit ripening.

Baninaiem et al. (2016) suggested that a combined preharvest and postharvest SA treatment is beneficial in extending the storability and hence SL of tomato fruit. In our study, endogenous SA is proposed to have a negative correlation with postharvest ET evolution and positive correlation with SL (Table 3), but this relationship depends on many aspects like the concentration of SA, antioxidative defense system, and oxidative stress in that line. The concentration-dependent role of exogenous SA has been reported by many research groups including ours (Fariduddin et al., 2003; Sayyari et al., 2009; Kant and Arora, 2014), and these groups also suggest that a very high level of exogenous SA application further reduces the SL of plant. A strong positive correlation of endogenous SA with SL at red stage in the present study accords with the published data; however, the concentration-dependent role of endogenous SA needs more evidence.

Detachment of fruit from plant affects its whole physiology including ET evolution (Bower et al., 2002). In the present study, the stage of detachment of fruit from plant and the internal environment of the line could be the reason of single peak in some and two peaks in other lines. The cultivar-specific role of internal as well as external environment is well-studied by Paul and Pandey (2014). The postharvest rise in ET level at red stage and more than one peak in some lines accord with the observations of Casas et al. (1990) and Van de Poel et al. (2012). Furthermore, postharvest treatment of kiwifruit with Acetyl salicylic acid (ASA) resulted in a reduced ACO and ACS activity and decreased ET production during the early stages of fruit ripening (Zhang et al., 2003). Our results suggested ET evolution working consistently with ACO in all the lines during postharvest fruit ripening. This observation was found to be in accordance with previously published data (Zhang et al., 2009). Wounding also stimulates ET evolution (Parsons and Mattoo, 1991) and ACS transcripts in tomato fruit, and there is a decline in wound-induced ACS by SA treatment (Li et al., 1992). The low levels of ACS in high SL lines (and high total endogenous SA) are in agreement with these reports.

Under abiotic stress, jasmonic acid (JA) is also reported to be working synergistically with ET biosynthesis (Lorenzo et al., 2003; Tamaoki et al., 2008). Plant resistance against pathogen depends on the synchronized ET–JA activation, which in turn makes plant insensitive toward SA-mediated suppression of JA-dependent defense (Leon-Reyes et al., 2009, 2010). Auxin response factors (ARFs) (Bouzroud et al., 2018) were also reported to be overexpressed against SA (Liu et al., 2018). Consequently, the whole process of ripening is a cross talk of various hormones, and a single phytohormone cannot regulate this process independently (Pérez-Llorca et al., 2019; Semeradova et al., 2020; Wang et al., 2020). Previous work from different research groups has shown evidence of hormone interaction in SA mutant studies during biotic and abiotic stress. SA mutant (NahG) analysis by López-Gresa et al. (2016) revealed very high levels of ET in SA-deficient plants upon viral infection in tomato fruit. Similarly, ABA treatment to flacca (ABA mutant) plants leads to SA and JA reduction in tomato under water stress (Muñoz-Espinoza et al., 2015). During fruit ripening, SA inactivates and it could interfere with the regulation of fruit ripening by influencing the ET levels. Mature green fruits (and not red ripe) are capable of SA-mediated response by NPR-1 (transcriptional co-regulator of SA responses and SA receptor in plants)-independent signaling pathway and can promote resistance to fruit against necrotrophic fungal infection (Blanco-Ulate et al., 2013). Data obtained in the present study showed antagonistic roles of endogenous SA and ET during postharvest fruit ripening, which accord with the published data of disease induction. But, contrary to previous work, this study showed the role of SA–ET antagonism at later stage (red) of fruit ripening instead of the earlier stages.

Another important observation in this study is that *SlICS* expression paralleled the accumulation of SA during the whole process of fruit ripening. The *PAL* gene family of tomato is very large with around 26 copies widely dispersed in the diploid genome, the majority of which is silenced (Chang et al., 2008). From the NCBI and TIGR database search, six *SlPAL* genes have been found in tomato. To determine tomato *SlPAL* and ripening relationship, expressions of *SlPAL4* and *SlPAL6* were studied. *SlPAL1* and *SlPAL5* have very low or no expression in tomato fruit, as suggested in previously published data (Guo and Wang, 2009; Gayoso et al., 2010). We were also unable to get a significant expression of *SlPAL2* and *SlPAL3* in tomato fruit during ripening. Previous studies of disease resistance in tomato roots suggested an increase in *PAL2, PAL3, PAL4*, and *PAL6* activity in resistant tomato varieties at different time points (Gayoso et al., 2010). A positively correlated increased *PAL5* and *PAL6* activities was found at low temperature and nitrogen-deficient conditions in tomato leaves (Løvdal and Lillo, 2009). Among eight rice *PAL* genes, *PAL2, PAL4*, and *PAL6* are found to be associated with disease resistance; however, there is a very complex interplay of PAL genes in rice upon disease infection (Lefevere et al., 2020). While studying tobacco PAL gene, Reichert et al. (2009) mentioned that there are multiple copies of PAL genes and they are differentially expressed among different plant tissues, which make any generalization about its role very difficult. However, in the present study of tomato fruits, *SlPAL6* is upregulated in low SL lines, while *SlPAL4* showed elevated expression in high SL lines. It indicates that ripening stimulates expression of genes for *PAL* rather than *ICS* evident by the positive association of *PAL4* with free SA during ripening. A similar type of finding with *PAL* gene was observed during chilling injury in cucumber seedlings (Liang and Shang, 2014).

### Antioxidative Defense

Plants have evolved various mechanisms to cope with the oxidative stress. The cellular damage by lipid peroxidation might be controlled or prevented by free radical and peroxide scavenging enzymes such as POX, SOD, CAT, GR, APX, etc. (Arora et al., 2002). In the present study, the positive correlation of TBARS with POX justifies the aforementioned statement. Here, most of the individual antioxidant activities significantly differed at both ripening stages due to lines, growing conditions, and environment effect. Significant differences were also found among lines and ripening stages without any definite pattern. In some lines, the levels of individual antioxidants rise from turning to red stage and, in others, they drop. Similar results were also previously observed by Cano et al. (2003); Meléndez-Martínez et al. (2010) in wild tomato varieties; García-Valverde et al. (2013) in four commercial tomato cultivars; and Bhandari and Lee (2016) in five cherry tomato and two general tomato cultivars, where individual antioxidants increased from breaker to red stage in tomato fruit. SA was found to be helpful in maintaining the higher levels of AA. In the present study, AA was observed to be low in high SL lines, suggesting that high SL fruit takes time to utilize AA hence delays the fruit softening and the consequent ripening. Hence, the results from this study accord with the previously published data regarding AA maintenance in postharvest SA-treated tomato (Pila et al., 2010), orange (Huang et al., 2008), pineapple (Lu et al., 2011), and rambutan (Supapvanich, 2015).

Orabi et al. (2015) suggested a crucial link between applied SA doses and antioxidant activities. In the present study, however, the total antioxidative capacity did not show any definite pattern with respect to the SA level (Figures 6H,I), but individual antioxidants did show some association (Figures 6D–H). It has been reported that application of exogenous SA increases fruit lycopene content (Javaheri et al., 2012); similarly, high endogenous SA content was found to be associated with high lycopene content, e.g., in DT-25 and Pusa Gaurav (Figures 6H,E). Lycopene also showed a positive correlation with SA at red stage (Table 3). Mandal et al. (2018) reported that postharvest treatment with SA (0.4 mM L^−1^) has the potential to prolong the storage life. However, contrary to our study, they concluded that SA shows a negative relationship with lycopene. In wheat, SA influenced other plant hormones like auxin, cytokinin, and abscisic acid and increased its growth and yield in normal as well as saline conditions (Fariduddin et al., 2003). In the present study, the association of lycopene content with SA could be ascribed to the influence of SA on other plant hormones. These effects also might be due to the promotion of ET synthesis at red stage, which contributes to changes in the level of lycopene (Zhu et al., 2015). Among antioxidants, the positive correlation of APX, POX, CAT, SOD, and GR with each other supports the fact that they work synchronously.

### Fruit Softening

Fruit softening is a main and critical quality change during fruit ripening that includes changes in cell wall integrity. Middle lamella is present on the outside wall of each cell, made up of pectic homogalacturonan molecules, which keeps the adjacent cells together. During ripening, homogalacturonan can be depolymerized by either pectate lyase (PL; by β-elimination, leaving a double bond at non-reducing end of the cleaved polysaccharide) or PG (by hydrolysis). Association of PG activity with fruit ripening is an established phenomenon now, although the amount varies from species to species (Hobson, 1962). In tomato, PG activity increases early in ripening (Smith et al., 1988a; Biggs and Handa, 1989) and continues to rise until fruit becomes overripe (Hobson, 1964; Tucker et al., 1980). In the present study, the increase in PG from turning to red stage supports the established phenomena. At turning stage, the positive relation of PG with TBARS confirms the importance of lipid peroxidation in cell wall degradation. Moreover, the negative correlation of PG with AA suggests the supportive role of AA in maintaining the cell wall integrity at red stage. However, the role of PG in fruit softening is still controversial (Brummell, 2020). The effect of suppression of two strawberry PG genes (*FaPG1* and *FaPG2*) on fruit firmness was not additive compared to their individual effect, and the suppression of one gene did not mean suppression of the other one, even though both encode PG (Paniagua et al., 2020). Silencing *PL* gene reduced cell wall softening, maintained fruit quality, increased SL, and decreased fungal pathogen susceptibility in tomato (Uluisik et al., 2016, Yang et al., 2017, Wang et al., 2019, Silva et al., 2020). So, for future studies, PL is a strong candidate for fruit firmness indicator.

PME plays very little role in ripening, but it does affect fruit senescence (Tieman and Handa, 1994). During tomato fruit ripening from mature green to red ripe, the degree of methyl-esterification of cell wall pectin declines from 90 to 35% (Koch and Nevins, 1989). Suppression of PME activity and consequently decreased pectin depolymerization did not affect fruit softening during normal ripening but in overripe fruits lead to an almost complete loss of tissue integrity (Tieman and Handa, 1994). Thus, PME activity plays little role in fruit softening but markedly affects tissue integrity during senescence (Brummell and Harpster, 2001). So, the decreasing trend of PME from turning to red in the present study was found to be consistent with the published data in tomato fruit. Moreover, the retention of fruit firmness as a result of SA treatment has been reported in many crops. Srivastava and Dwivedi (2000) reported that in SA-treated banana fruit, softening significantly decreased methyl salicylic acid (MeSA), in a concentration-dependent manner, and maintained kiwifruit firmness during storage (Aghdam et al., 2009). It has been stated that SA treatment inhibits cell wall and membrane-degrading enzymes such as PG and PME and reduces ET production, leading to decreased fruit softening rate (Srivastava and Dwivedi, 2000; Zhang et al., 2003; Kant and Arora, 2014). A positive correlation between the fruit's free SA content and kiwifruit firmness during ripening was also reported by Zhang et al. (2003). Here also, a positive correlation was found between endogenous SA and PME at red stage (Table 3). But the strong negative correlation and strong positive correlation of PME with ET and lycopene, respectively, at red stage suggest that PME plays its role as ripening advances. So, high PG decreases cell wall integrity; on the other hand, PME does not affect fruit softening, but it has a role in reducing ripening. Furthermore, PME could be regulated by JA (Bethke et al., 2016). Recently, upon postharvest SA application in kinnow mandarin, it was found that this treatment affected the enzymatic and total antioxidative capacity but does not influence the carotenoid, pectic substances or fruit-softening enzyme activity (Haider et al., 2020); so, our report agrees with this recent report at enzymatic antioxidant and PME levels.

MSI represents the change in the permeability of the membrane. Reduction in MSI indicates the status of ion leakage or solute leakage. As fruit ripening progresses, there is a sharp decline in MSI in all the lines studied (Figure 2B). Here, the positive correlation of MSI with SL confirms that high MSI is an important attribute to high SL.

## Conclusion

The present study suggests that endogenous SA has a positive correlation with SL, and it regulates postharvest ET production (Table 3). But it works in a concentration-dependent manner, and its optimal concentration to reduce the ripening process varies from cultivar to cultivar and variety to variety in tomato fruit. CF of more than 600 was found in high SL lines. Along with *SlICS*, SA pathway gene *SlPAL4* is also associated with high SL, so it could serve as a potential target to delay ripening. The antioxidative defense also plays a major role in reducing the oxidative stress and increasing the SL of fruit especially at the level of SOD, CAT, GR, POX, and lycopene. PME also affects ET production in high lycopene lines. But, above all, it is the genetic potential of the lines and then the plant itself that decides its ripening-related phenomenon. In the future, molecular and functional analyses of downregulated and/or overexpressed SA pathway gene(s) can be done to better understand the underlying mechanism of SA-associated postharvest fruit ripening. To better understand the SA–ET interaction, mutant studies could also be done.

## Data Availability Statement

The original contributions presented in the study are included in the article/Supplementary Material, further inquiries can be directed to the corresponding author/s.

## Author Contributions

AA proposed the hypothesis and conceived the idea. CC and TS performed the experiments and data analysis. CC wrote the manuscript. CC, MA, and AA finalized the manuscript and all authors approved it. ZH grew plants in the field and provided tomato fruit. NS helped in optimizing SA extraction protocol for HPLC. AK and VS helped in improvisation of methodology. All authors contributed to the article and approved the submitted version.

## Conflict of Interest

The authors declare that the research was conducted in the absence of any commercial or financial relationships that could be construed as a potential conflict of interest.

## Abbreviations

SA: salicylic acid
ET: ethylene
SL: shelf life
JA: jasmonic acid
PG: polygalacturonase
PME: pectin methyl esterase
MSI: membrane stability index
*ICS*: *IsoChorismate synthase*
*PAL*: phenylalanine ammonia lyase
POX: peroxidase
SOD: superoxide dismutase
CAT: catalase
GR: glutathione reductase
APX: ascorbate peroxidase
AA: ascorbic acid.
