# Supplementary material for: Regulation of Postharvest Tomato Fruit Ripening by Endogenous Salicylic Acid
Source: Front Plant Sci. 2021 Jun 7;12:663943. doi: 10.3389/fpls.2021.663943 (PMC8216237; doi:10.3389/fpls.2021.663943)
Supplement: Supplementary file 1 [file Table_1.docx]

| **Supp. table 1. List of lines studied.** | | |
| --- | --- | --- |
| **S. No.** | **Genotype ID** | **Plant type and special features** |
|  | DT-36 | Pre-released pure line, Determinate; fruit medium (70g), oblong, uniform ripening and High TSS (5.2 ^0^B) |
|  | DT-37 | Imported line, Indeterminate; fruit medium (60g), round, uniform ripening and TSS (4.2 ^0^B) |
|  | DT-38 | Pre-released pure line, Determinate; fruit medium (70g), oblong, uniform ripening and good for long distance transportation |
|  | DT-30 | Pre-released pure line, Determinate; fruit medium (80g), round, uniform ripening and good for long distance transportation. |
|  | Pusa Sadabahar | IARI released Indian cultivar, Determinate, thermo-tolerant, fruit set takes place almost round the year, both hot and cold set. |
|  | DT-39 | Imported germplasm line, Indeterminate; fruit medium (60g), oblong, dark red coloured, uniform ripening and TSS (4.2 ^0^B) |
|  | DT-40 | Released Indian cultivar, Indeterminate; fruit medium (70g), oblong, uniform ripening and TSS (4.8 ^0^B) |
|  | Punjab Tropic | PAU released variety, Plants determinate; fruit medium (80g), round, uniform ripening and good for long distance transportation. |
|  | DTH-32 | Pre-released hybrid, Indeterminate; fruit medium (60g), oblong, uniform ripening and TSS (4.0 ^0^B) |
|  | DT-41 | Pre-released pure line, Determinate; small fruit (60g), oblong, uniform ripening and TSS (5.0 ^0^B) |
|  | DT-42 | Imported germplasm line, Dwarf, Determinate; small fruit (50g), oblong, uniform ripening and TSS (5.2 ^0^B) |
|  | Pusa Ruby | IARI released variety, Indeterminate, High temperature tolerance, fruit flat, red, TSS (5.1 ^0^B) |
|  | DT-43 | Imported exotic variety, Determinate; small fruit (60g), cylendrical, uniform ripening and TSS (5.0 %) |
|  | DT-44 | Imported exotic variety, Indeterminate Oblong fruit, TSS (5.2 ^0^B). |
|  | PH-8 | Released Indian hybrid, IARI released hybrid, Plants determinate; fruit medium (75-80g), round, uniform ripening. |
|  | DTH-33 | Released exotic hybrid, Determinate, dark green, fruit red, spherical, fruit weight of 90 g. |
|  | DT-45 | Released cultivar, Determinate, dark green, fruit red, spherical, size medium to large, weight of 80 g. |
|  | DT-46 | Imported exotic variety, Determinate, fruit red, spherical, size medium to large, weight of 60 g. |
|  | DTH-47 | Released Indian cultivar, Indeterminate; fruit medium (90g), flat, potato leaf type, uniform ripening and TSS (4.9 ^0^B) |
|  | DTH-34 | Released hybrid, Plants determinate; fruit medium (75-80g), round, uniform ripening. |
|  | DVRT-2 | Released Indian cultivar, Determinate; fruit medium (60g), round, potato leaf type, uniform ripening and TSS (4.9 ^0^B) |
|  | DT-48 | Pre-released pure line, Determinate; fruit medium (60g), oblong, uniform ripening and TSS (4.6 ^0^B) |
|  | DT-49 | Released Indian cultivar, Indeterminate; fruit medium (60g), oblong, uniform ripening and TSS (5.0 ^0^B) |
|  | DTH-45 (21) | Hybrid variety released by ICAR-IIHR, Bangalore, India. Plants semi-determinate with dark green foliage. Fruit oblate, medium large (80-90 g.) with light green shoulder. Suitable for table purpose. |
|  | DT-25 (21) | Breeding line, Plants determinate; fruit medium (80g), round, uniform ripening and good for long distance transportation |
|  | Pusa Hybrid-4 | IARI released Indian hybrid, Determinate, Fruit are attractive round having thick skin and uniform ripening. Average weight is 70-80 g this is suitable for the areas having problem of rootknot nematode. |
|  | Pusa Gaurav | IARI released Indian variety, Plants determinate; fruit medium (60g), round, uniform ripening and good for long distance transportation. Good for processing. |
|  | DT-51 | Pure line, Determinate, fruit medium (70 g), round, uniform ripening, round, TSS (5.1 ^0^B) |
|  | Punjab Upma | Released Indian cultivar, Determinate, fruit medium (70 g), oblong, uniform ripening, round, TSS (5.0 ^0^B) |
|  | DTH-25 | Pre-released hybrid, Determinate; fruit medium (70 g), round, uniform ripening and good for long distance transportation. |
|  | DT-52 | Released Indian cultivar, Determinate, dark green, fruit red, spherical, size medium to large, weight of 80 g. |
|  | DTH-37 | Pre-released hybrid, Determinate, fruit medium (70 g), round, uniform ripening and good for long distance transportation. |
|  | DT-53 | Homozygous pure line, Indeterminate, fruit wt. (60 g), fruit dark red, oblong. |
